# The effect of depression on occupational efficacy of higher vocational college students: the mediating role of psychological resilience and social support

**DOI:** 10.3389/fpsyt.2025.1470204

**Published:** 2025-07-25

**Authors:** Li Sun, Yeqing Zhang, Hongtao Xie, Qingwei Li, Zhipei Zhu

**Affiliations:** ^1^ Academic Center for Disease Control and Mental Health, Shanghai Putuo Mental Health Center, Shanghai, China; ^2^ Shanghai Mental Health Center, Shanghai Jiao Tong University School of Medicine, Shanghai, China

**Keywords:** social support, psychological resilience, occupational efficacy, depression, higher vocational college students

## Abstract

**Objective:**

To understand the current situation of vocational college students’ mental health, take depression as independent variable, and explore the mechanism of social support and mental toughness as mediating variables on vocational college students’ career efficacy, so as to provide basis for effectively improving teenagers’ career efficacy, promoting teenagers’ mental health and future development.

**Methods:**

A total of 780 students in the first and second grades of high school in vocational schools were selected as the research objects by random cluster sampling. The questionnaire survey was conducted by the 9-item Patient Health Questionnaire (PHQ-9), Resilience Scale for Chinese Adolescents (RSCA), Social Support Rate Scale (SSRS) and career decision self-efficacy scale of secondary vocational students.

**Results:**

Depression was negatively correlated with occupational efficacy, mental toughness and social support (*P* < 0.01). Psychological resilience was negatively correlated with occupational efficacy (*P* < 0.01), while social support was positively correlated with occupational efficacy (*P* < 0.01). The influence of mental toughness on occupational efficacy showed a masking effect, and the indirect effect accounted for 18.90% of the direct effect. Social support plays a partial mediating role in the effect of depression on occupational efficacy, accounting for 35.87% of the total effect.

**Conclusion:**

Psychological resilience shows masking effect in the influence of depression on occupational efficacy, which is limited and influenced by personal cognition, and emphasizes the importance of emotional management. Social support plays an important role in reducing adolescent depression and improving adolescent occupational efficacy, and can effectively alleviate the adverse effects of depression on adolescent occupational efficacy. It is suggested that family, school and individual efforts should be made to strengthen the social support network, improve the psychological resilience of adolescents, and help improve their mental health level and professional efficacy.

## 1 Introduction

Adolescence is a critical period of cognitive and personality maturity, self-consciousness differentiation, self-contradiction intensifies, and physical and mental development presents a significant imbalance, which is a high incidence period of psychological problems (1). Under the influence of external factors such as intense employment competition, academic burden, peer pressure and parental control, vocational college students are in a relatively high state of psychological stress. When individuals make unreasonable evaluation of self-ability or have unreasonable expectations for their life goals, internal conflicts increase significantly, stress response increases, and depression and anxiety are more likely to occur (2). Recent studies have found that depression is an important risk factor for mental health. Family heredity, family environment, parenting style, coping style and personality traits may be the precipitating factors of depression. It not only affects the individual’s quality of life and social adaptation level, but also is closely related to the sense of professional efficacy, causing great harm to the society (3, 4).

American psychologist Bandura proposed the concept of “self-efficacy” (5), which is a kind of overall self-confidence that an individual has when facing a new environment or new things. Research found that self-efficacy was negatively correlated with depression, state anxiety and trait anxiety, and positively correlated with emotional intelligence and positive coping styles (6). Career efficacy, also known as career decision self-efficacy, is the ability and belief necessary for an individual to complete tasks related to a career (7), which determines an individual’s participation, investment and firmness in career activities. The level of career efficacy directly or indirectly affects an individual’s career choice, thus affecting the development of individual potential and the realization of career goals (8). Zhou Jian et al. found that career efficacy is significantly positively correlated with the employment quality of higher vocational students (9). Compared with personal background, career efficacy has a more prominent impact on the employment quality of higher vocational students.

Psychological resilience is the ability of an individual to recover quickly and overcome the damaging effects of severe stress or adversity. Psychological resilience plays a protective role in coping with external pressure and psychological problems, and its good development helps individuals avoid the influence of risk factors and maintain their mental health (10). Social support refers to the sum total of free help to socially vulnerable groups by a certain social network using certain material and spiritual means. It usually refers to the system that provides spiritual or material help and support to individuals from various aspects of society, including parents, relatives, friends and so on (11). Good social support can improve individual social adaptability, and play an important role in maintaining individual mental health and buffering the adverse effects of psychological stress.

Individuals with different personality traits respond differently to the same external pressures. Foreign studies have proved that fragile psychological quality can become a catalyst for extreme emotions and behaviors of individuals (12). At present, most of the existing studies focus on the influence of family environment on adolescent depression and career effectiveness, and pay less attention to vocational college students. This study takes vocational college students as the research object, with depression as the independent variable, psychological resilience and social support as the mediating variable, and career efficacy as the dependent variable. It is of great theoretical and practical significance to establish the mediating effect model and explore the internal mechanism of psychological resilience and social support in the relationship between depression and occupational efficacy, which is helpful to improve the early identification and intervention of adolescent depression and enhance vocational college students’ occupational efficacy.

## 2 Methods

### 2.1 Participants

From December 1 to December 15, 2022, random cluster sampling was used to select Grade 1 and Grade 2 high school students in a district-level vocational school in Shanghai as the research objects. Inclusion criteria: age 16 ~ 18 years old; Be able to participate in school studies and activities; Normal understanding, ability to communicate normally and fully express their feelings; During the investigation, the emotional state was stable and the consciousness was clear; Informed consent, voluntary participation in this study. Exclusion criteria: severe negativity, mania, autism, intellectual disability, borderline personality disorder and other significant physical and mental illness can’t cooperate with the investigation; Severe brain and physical disorders and psychoactive substance abuse. A total of 813 questionnaires were issued, and 780 valid questionnaires were collected, with an effective rate of 95.94%. There were 780 valid subjects, including 485 males (62.18%), 295 females (37.82%), 460 first-year vocational high school students (58.97%) and 320 second-year vocational high school students (41.03%).

### 2.2 Process and quality control

Trained psychiatrists and school psychology teachers formed investigation teams, using uniformly printed self-designed questionnaires and standardized measurement scales. After the unified training, the school psychology teacher, under the coordination of the class teacher, conducted a group test in class as a unit, and collected the questionnaire immediately after filling in. All respondents filled in the questionnaire voluntarily, and students and parents signed the informed consent. As the main test, the psychology teacher needs to explain the guiding words in detail, explain the significance of the investigation, and require the students to answer independently according to their actual situation. The questionnaire was filled in anonymously, and all questions were required.

### 2.3 Measures

#### 2.3.1 Sociodemographic variables

Sociodemographic variables included gender and age. In order to protect the privacy of young students, personal name and contact information were not involved.

#### 2.3.2 Depression symptoms

The Patient Health Questionnaire-9 (PHQ-9) was adopted to assess depression symptoms during the past two weeks (13). There are 9 items and each item is rated from 0 (not at all) to 3 (nearly every day), and higher total score indicates more severe depressive symptoms. The Cronbach’s α was 0.922.

#### 2.3.3 Occupational efficacy

The Scale of Self-efficacy of Career decision Making for secondary vocational Students (14) was compiled and revised by Peng and Long (14), and modified by Pan Y, (15) into a scale of career decision making self-efficacy suitable for secondary vocational students, including 39 items in 5 dimensions such as self-evaluation, information collection, goal selection, planning and problem solving. Likert 5-point scale is used to score points, and the higher the score, the stronger the self-efficacy of career decision making. The Cronbach’s α was 0.886.

#### 2.3.4 Psychological resilience

Resilience Scale for Chinese Adolescents (RSCA) was developed by Hu Yueqin et al. (16), which was used to measure the psychological resilience of adolescents in the face of life adversity and trauma with major pressures. There are 5 dimensions in total, of which 3 factors (goal focus, emotional control and positive cognition) are classified as individual dimension, and 2 factors (family support and interpersonal assistance) are classified as supportive dimension. There are 27 items in the scale, and the Likert 5-point scale is used to score points. A higher score indicates greater psychological resilience. The Cronbach’s α was 0.914.

#### 2.3.5 Social support

The Social Support Rate Scale (SSRS) was compiled by Xiao Shuiyuan et al. (17), with a total of 10 items, 3 of which belong to the objective support dimension (2/6/7), 4 of which belong to the subjective support dimension (1/3/4/5) and 3 of which belong to the social support utilization dimension (8/9/10). The total score is the sum of the scores of ten items, and the higher the score, the higher the social support. The Cronbach’s α was 0.891.

### 2.4 Statistical analysis

All questionnaires were recorded by two persons and SPSS 24.0 was used for statistical analysis. Continuous variables were represented as mean ± standard deviation, and independent sample t-test was used to test the difference. The categorical variable was expressed as percentage and Chi-square test was used to test the difference. Spearman correlation analysis was used for the correlation between the two variables, and AMOS 24.0 was used to establish, revise and evaluate the structural equation model to test the mediation effect. *P* < 0.05 was considered statistically significant.

## 3 Results

### 3.1 Correlation analysis of each scale

PHQ-9 was negatively correlated with occupational efficacy scale, RSCA and SSRS (*P* < 0.01). There was a significant negative correlation between RSCA and occupational efficacy scale (*P* < 0.01), and there was a significant positive correlation between SSRS and occupational efficacy scale (*P* < 0.01), as shown in **Table 1**. In the professional efficacy scale, the scores of the five factors of self-evaluation, information collection, goal selection, plan making and problem solving were significantly negatively correlated with the two factors of goal focus and positive cognition in the RSCA scale, and were significantly positively correlated with the two factors of emotional control and interpersonal assistance. The scores of 5 factors in the occupational efficacy scale were significantly positively correlated with the three factors of objective support, subjective support and social utilization in the SSRS scale (*P* < 0.01), as shown in **Table 2**.

**Table 1:** Correlation analysis of each scale score.

	PHQ-9	RSCA	SSRS	Occupational efficacy
PHQ-9	1			
RSCA	-0.323^**^	1		
SSRS	-0.288^**^	0.005	1	
Occupational efficacy	-0.286^**^	-0.144^**^	0.363^**^	1

**p < 0.01.

**Table 2:** Correlation analysis among the factors of the scales.

		Occupational efficacy				
		Self-evaluation	Information collection	Goal selection	Plan making	Problem solving
RSCA	goal focus	-0.452^**^	-0.436^**^	-0.414^**^	-0.432^**^	-0.411^**^
	emotional control	0.222^**^	0.224^**^	0.229^**^	0.183^**^	0.183^**^
	positive cognition	-0.318^**^	-0.339^**^	-0.310^**^	-0.333^**^	-0.326^**^
	family support	-0.019	-0.028	-0.018	-0.044	-0.025
	interpersonal assistance	0.097^**^	0.115^**^	0.147^**^	0.105^**^	0.118^**^
SSRS	objective support	0.160^**^	0.195^**^	0.201^**^	0.210^**^	0.189^**^
	subjective support	0.306^**^	0.348^**^	0.334^**^	0.307^**^	0.274^**^
	social utilization	0.297^**^	0.298^**^	0.294^**^	0.290^**^	0.273^**^

**p < 0.01.

### 3.2 The mediating effect of psychological resilience on the influence of depression on occupational efficacy

**Figure 1:**
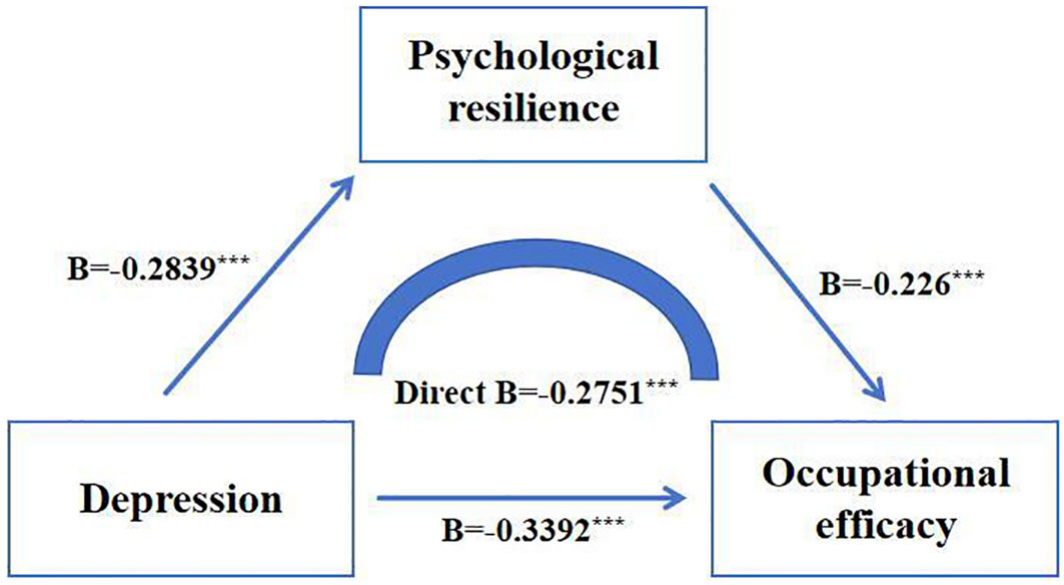
The mediating model of psychological resilience.

In Model 1, with occupational efficacy as the dependent variable and depression as the predictor, the results showed that depression significantly negatively predicted occupational efficacy(*β* = -0.2751, *p* < 0.001), which could explain 7.57% of the variation of the dependent variable. In Model 2, with mental toughness as the dependent variable and depression as the predictor, the results showed that depression significantly negatively predicted mental toughness (*β* = -0.2839, *p* < 0.001), which could explain 8.06% of the variation of the dependent variable. In model 3, when depression and mental toughness predicted occupational efficacy at the same time, the negative prediction effect of mental toughness on occupational efficacy was significant (*β* = -0.226, *p* < 0.001), and the negative prediction effect of depression on occupational efficacy was still significant (*β* = -0.3392, *p* < 0.001), 12.26% of the variation in the dependent variable can be explained jointly. The mediating effect is 0.0641. Psychological resilience has a masking effect on the influence of depression on occupational efficacy, and the indirect effect accounts for 18.90% of the direct effect, as shown in **Figure 1**, **Table 3**.

**Table 3:** Analysis of the mediating effects of psychological resilience.

	Model 1		Model 2		Model 3	
	Y = Occupational efficacy		M = Psychological resilience		Y = Occupational efficacy	
	B	SE	B	SE	B	SE
X = PHQ-9	-0.2751^***^	0.0345	-0.2839^***^	0.0344	-0.3392^***^	0.035
M = Psychological resilience					-0.226^***^	0.035
*F*	63.70^***^		68.189^***^		54.297^***^	
*R^2^*	0.0757		0.0806		0.1226	
Sobel Test	Indirect effect = 0.0641		Z = 5.059^***^			
Bootstrap	Indirect effect 0.0641		BootLLCI 0.0329		BootULCI 0.1001	
		

***p < 0.001.

### 3.3 The mediating role of social support in the influence of depression on occupational efficacy

**Figure 2:**
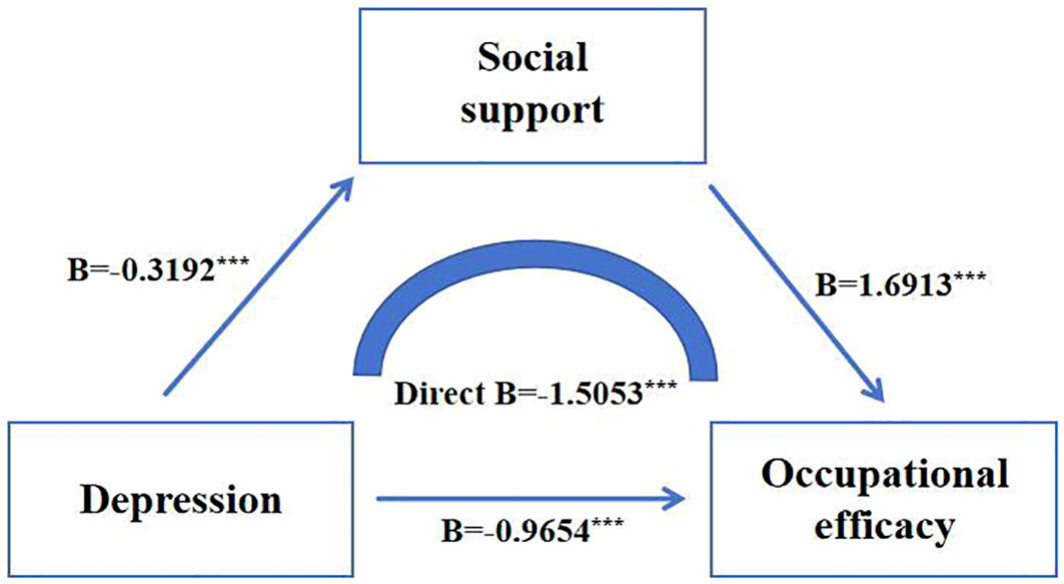
The mediating model of social support.

In Model 1, with occupational efficacy as the dependent variable and depression as the predictor, the results showed that depression significantly negatively predicted occupational efficacy (*β* = -1.5053, *p* < 0.001), which could explain 7.57% of the variation of the dependent variable. In Model 2, with social support as the dependent variable and depression as the predictor, the results showed that depression significantly negatively predicted social support (*β* = -0.3192, *p* < 0.001), which could explain 9.40% of the variation of the dependent variable. In model 3, when depression and social support predicted occupational efficacy at the same time, social support had a significant positive predictive effect on occupational efficacy (*β* = 1.6913, *p* < 0.001), while depression had a significant negative predictive effect on occupational efficacy (*β* = -0.9654, *p* < 0.001). It could jointly explain 16.95% of the variation in the dependent variable, and the mediating effect was 0.5399. Among the effects of depression on occupational efficacy, social support showed a partial mediating effect, and the mediating effect accounted for 35.87% of the total effect, as shown in **Figure 2**, **Table 4**.

**Table 4:** Analysis of the mediating effects of social support.

	Model 1		Model 2		Model 3	
	Y = Occupational efficacy		M = Social support		Y = Occupational efficacy	
	B	SE	B	SE	B	SE
X = PHQ-9	-1.5053^***^	0.1886	-0.3192^***^	0.0355	-0.9654^***^	0.1879
M = Social support					1.6913^***^	0.1805
*F*	63.6995^***^		80.703^***^		79.306^***^	
*R^2^*	0.0757		0.094		0.1695	
Sobel Test	Indirect effect = -0.5399		Z = -6.4656^***^			
Bootstrap	Indirect effect -0.5399		BootLLCI -0.7441		BootULCI -0.3748	
		

***p < 0.001.

## 4 Discussion

Vocational college students’ cognitive ability has gradually developed and improved. Due to the influence of stressful events such as career selection and the instability of their own hormone level, their psychological state is prone to drastic changes, and they often experience the emotional arousal of high pressure level. High intensity emotional arousal can induce anxiety, depression, depression, boredom and other adverse emotional experiences, which makes vocational college students’ mental health problems become increasingly prominent, and they become high-risk groups of depression. Some individuals have self-harm and suicide behaviors, which seriously affect life and study (18). In this study, a mediating effect model was established to explore the internal mechanism of psychological resilience and social support in the influence of depression on occupational efficacy.

### 4.1 Correlation analysis between depression and psychological resilience, social support and occupational efficacy

The results of this study show that vocational college students’ depression is significantly negatively correlated with mental toughness, social support and career efficacy, and mental toughness is significantly negatively correlated with career efficacy. It shows that vocational college students with depression have poor mental toughness and social support, low career efficacy and decreased career confidence. Poor mental toughness, reduce individual emotional control ability, the ability to buffer the impact of negative life events is insufficient, easy to produce negative, self-directed negative automatic thinking (19). At the same time, the interaction between negative thinking and the external environment affects the individual’s self-cognitive ability, resulting in a plummeting level of psychological resilience of patients, resulting in negative self-evaluation, withdrawal, avoidance and other behaviors, resulting in an increased level of depression, and lower confidence in coping with occupational pressure and successful employment. When in a stressful environment, individuals are prone to accumulate depression and anxiety, increasing the risk of depression (20, 21). Combined with the stress-susceptibility model, the group with low mental toughness is indeed a vulnerable group to academic stress problems, and they are more likely to have strong depression and suicidal thoughts (22). The higher the psychological resilience, the more positive the individual’s emotional cognition, emotional regulation and control, which can help adolescents cope with pressure in a positive way, have a stronger positive recovery from adversity, produce the ability to cope with stressful events, successfully adapt to setbacks, reduce the occurrence of depression, and inhibit the subsequent damage of negative emotions (23, 24).

Social support includes providing emotional support, material support, dealing with realistic problems in life, and providing accessible and effective information, social networks and other positive social relationships to protect the mental health function of individuals by creating a safe and supportive environment for individuals. This study found that vocational college students’ social support has a significant negative correlation with depression, and a significant positive correlation with career efficacy. When adolescents lack effective social support, they find it difficult to cope with failure, have no confidence in success again, and are prone to academic burnout such as anxiety, compulsion and depression (25). Insufficient social support is also related to academic adjustment problems, peer rejection, and impulsive behaviors (26). Cheong et al. found that the lack of social support in childhood increases the probability of depression, indicating that social support has a protective effect. When individuals lack social support, stress resistance decreases and depression is more likely (27). Watanabe et al. found that accessible social support, such as professional help objects and methods, is an important link in reducing the risk of self-injury and suicide caused by depression (28). Empirical studies have also confirmed that teenagers raised by their grandparents are prone to neglect, lack of spiritual and material support when they encounter problems, and cannot get timely attention and guidance, and depression and other negative emotions occur frequently (29).

Individual psychological development mainly depends on the support of external resources or protective factors. Psychological resilience is an indicator of the ability to resist pressure, while social support is a very important external resource. Both psychological resilience and social support can be involved in the regulation of depression, and are closely related to the formation of career efficacy, which further affects the career choice and development of adolescents. Under the influence of depression, teenagers often feel that they cannot adapt to the workplace environment, are confused about career choice, full of anxiety and fear, and lack of planning and confidence in future career development, which leads to uncertainty and confusion in career development and affects personal career development (30). Zeng Rongxia found that adolescents’ self-efficacy was negatively correlated with depression and anxiety, and positively correlated with emotional intelligence and positive coping styles (3). Therefore, attention should be paid to the influence of depression on adolescents’ occupational efficacy, and timely measures should be taken to actively reduce the occurrence of depression and improve adolescents’ occupational ability and ability to plan their career future.

### 4.2 The mediating effect of psychological resilience on the influence of depression on occupational efficacy

The results show that there is a significant negative correlation between vocational college students’ mental toughness and career efficacy. Among the effects of depression on occupational efficacy, psychological toughness has a masking effect. Adolescents are in the slow formation stage of the psychological resilience structure, and the more setbacks they experience, the more personality traits such as goal-focused and positive cognition will develop. Although the higher the psychological resilience of adolescents in coping with adversity or pressure, the more positive the individual’s emotional cognition, emotional regulation and control will be, the impact of setbacks and adverse events has not been effectively eliminated. Accompanied by depression, the degree of self-acceptance is low, which further affects the development of professional efficacy. Studies have found that adolescents with high psychological resilience have self-regulation to form internal thinking and motivation for positive coping, view negative events with a calm mind, better emotional control in the face of academic and occupational pressure, better interpersonal assistance ability, often have positive job-hunting behaviors, easier to make career decisions and succeed in employment, and have more confidence in their career. However, this is only a high starting point advantage brought about by the development of psychological quality, and it is speculated that it may be related to cognitive bias rather than protective effect. Similar findings were found in Wu et al. (31).

Studies have confirmed that a good social support environment is conducive to psychological training, in which the improvement of mental toughness is an important manifestation, thus stimulating more positive behaviors of adolescents (32). Family is an important basic factor to cultivate adolescent psychological resilience, including parental encouragement, support, help and guidance, etc. A good family atmosphere and parenting style are also positive factors to improve psychological resilience, guide to change negative self-cognition and other personality traits, help individuals to learn to obtain favorable resources to promote growth, and eliminate the negative impact on the long-term development of higher vocational students.

In addition, the mediating role of psychological resilience emphasizes the importance of emotional management. Teaching adolescent positive emotion management strategies, such as changing perspective and cultivating optimistic future orientation, can change adolescents’ negative self-cognition in the face of failure and reduce the impact of negative events on professional efficacy. Studies have confirmed that a good supportive environment is conducive to improving psychological resilience, thus stimulating more positive behaviors among adolescents Family is an important basic factor to train the psychological resilience of adolescents, and a good family atmosphere and parenting style are positive factors to improve psychological resilience, which can help individuals to obtain favorable resources and promote growth.

### 4.3 The mediating effect of social support on the influence of depression on occupational efficacy

The results of this study show that social support is significantly positively correlated with career efficacy and has a significant positive predictive effect on career efficacy, indicating that adolescents with stronger social support are more confident in career choice. Liu Chunhui also found that social support can improve adolescents’ self-efficacy and career maturity (33). The mediating model analysis showed that social support had a partial mediating effect on the significantly negative prediction of career efficacy of depression. With relatively sufficient social support and a relaxed growth environment, teenagers are easy to form a lively, cheerful and trusting personality. When they encounter problems, they can communicate with their parents to solve problems in time, improve their ability to cope with bad emotions, enhance their ability to cope with academic pressure, and have high achievement motivation, which plays a positive regulating role in career efficacy (34) and resists the negative influence of depression on occupational efficacy. In addition, the successful experience of using social support systems has enhanced the confidence of regulating negative emotions such as depression, which can effectively buffer the adverse consequences of occupational stress.

The emotional support of family members is an important positive factor for adolescents’ independent development. Positive parent-child communication can improve teenagers’ problem-solving ability and play an important role in their career development (35). Good family intimacy provides a good atmosphere for the healthy physical and mental development of adolescents (36). Parents’ effective participation and support in young people’s study, life, interpersonal communication, and school affairs can help young people improve their ability to cope with all kinds of bad emotions, cultivate psychological resilience, and enhance career achievement. On the contrary, family dysfunction, adolescents’ emotional needs are easily ignored, they are prone to insecurity when facing pressure, lack of reasonable coping methods, and increase the risk of academic burnout and depression. Therefore, it is necessary to strengthen the support of family, school, relatives and society to teenagers, reduce the influence of bad emotions, enhance happiness, and promote the formation of professional efficacy.

## 5 Conclusion

This study explores the mechanism of the influence of psychological resilience and social support on vocational college students’ career efficacy through the mediation effect model. Psychological resilience shows masking effect in the influence of depression on occupational efficacy, which is limited and influenced by personal cognition, and emphasizes the importance of emotional management. Social support plays an important role in reducing adolescent depression and improving adolescent occupational efficacy, and can effectively alleviate the adverse effects of depression on adolescent occupational efficacy.

## 6 Suggestion

In order to improve the mental health level of adolescents, on the one hand, it is necessary to guide and change their negative self-cognition, and on the other hand, it is necessary to use a powerful social support system to cultivate and shape the psychological resilience of adolescents (37). It is necessary to help teenagers deal with pressure and cope with external adverse events in a timely manner, pay attention to emotional education and cognitive coping education for teenagers, enrich their social resources, improve the cultivation of individual personality characteristics such as emotional regulation ability, reduce the occurrence of depression, and make teenagers more confident in employment and more adaptable to adversity (38). Efforts are made to improve the parenting style of parents, increase the time of parent-child companionship, especially the companionship of fathers, and strive to create a democratic and tolerant family atmosphere; Cultivate the positive psychological quality of adolescents, improve the level of resilience to cope with negative events, and reduce the level of depression; The campus should increase the corresponding mental health lectures, and provide channels for campus psychological counseling and crisis management; Encourage and guide adolescents to participate in social activities, establish more social relationships with others, and provide a supportive environment for the whole society (39). We need to increase the channels to provide social support through various ways to help teenagers fully understand themselves, improve self-efficacy, develop their own potential, actively explore, and correctly choose the future career direction in the constant adjustment, and help them improve their professional efficacy.

## 7 Limitations

At present, most studies on career efficacy take single factor as independent variable, and few studies explore the mediating role of multiple factors. The conclusion of this study provides a new idea for the promotion mechanism of career efficacy. At the same time, there are limitations. The first and second grades in higher vocational schools are selected, and the sample representation is insufficient. Our study could not exclude the influence of gender and age on the results. The follow-up study will further expand the study area and study population to obtain more reliable conclusions.

## Data Availability

The original contributions presented in the study are included in the article/Supplementary Material. Further inquiries can be directed to the corresponding authors.

## References

[1] Zhang XM Qi M Li HJ Lin JY Liu HQ Chen JX. The relationship between life events and depression in middle school students: the mediating role of cognitive flexibility. J Clin Psychiatry (Chin). (2020) 30:393–7.

[2] Lin H Huang QK Tong SL. Effects of family functioning and stress on adolescents’ mental health. Chin J Child Health Care (Chin). (2022) 30:1380–4.

[3] Zeng RX Wu W. A study on the relationship between general self-efficacy, coping style and depression among college freshmen. J Henan Institute Educ (Chin). (2008) 2):109–10.

[4] Song WH Chen Y Ma XZ Bai YX. The correlation between mental toughness and depression in vocational college students: a comparison between poverty and poverty. J Zhejiang Polytech Industry Trade (Chin). (2020) 20:16–21.

[5] Bandura A. Self-efficacy: The exercise of control. New York, NY, US: W H Freeman/Times Books/Henry Holt & Co (1997).

[6] Zhang LL Yang Y Wang GS Chen HB Wang SL Yin WJ. Mental health status and related factors of adolescents with NSSI. J Psychiatry (Chin). (2022) 35:139–45.

[7] Feehan PF Johnston JA. The self-directed search and career self-efficacy. J Career Assess. (1999) 7:145–59. doi: 10.1177/106907279900700204

[8] Gureje O Uwakwe R Oladeji B Makanjuola VO Esan O. Depression in adult Nigerians: results from the Nigerian Survey of Mental Health and Well-being. J Affect Disord. (2010) 120:158–64. doi: 10.1016/j.jad.2009.04.030, PMID: 19450883

[9] Zhou J. A study on the influence of career efficacy on vocational students’ employment quality – A case study of Guizhou D Vocational College (chinese). (Master). (2021).

[10] Rainey EE Petrey LB Reynolds M Agtarap S Warren AM. Psychological factors predicting outcome after traumatic injury: the role of resilience. Am J Surg. (2014) 208:517–23. doi: 10.1016/j.amjsurg.2014.05.016, PMID: 25124293

[11] Cohen S Wills TA. Stress, social support, and the buffering hypothesis. Psychol Bull. (1985) 98:310–57. doi: 10.1037/0033-2909.98.2.310 3901065

[12] Johnson J Wood AM Gooding P Taylor PJ Tarrier N. Resilience to suicidality: the buffering hypothesis. Clin Psychol Rev. (2011) 31:563–91. doi: 10.1016/j.cpr.2010.12.007, PMID: 21276646

[13] Hu XC Zhang YL Liang W Zhang HM Yang SC. Reliability and validity of Patient Health Questionnaire Depression Scale (PHQ-9) in adolescents. Ment Health Sichuan (Chin). (2014) 27:357–60.

[14] Peng YX Long LR. Self-efficacy assessment of college students’ career decision making. Appl Psychol (Chin). (2001) 7(2):45–7.

[15] Pan Y. The Relationship between career decision-making self-efficacy of secondary vocational school students and development resources as well as academic performance (chinese). (Master Thesis) (2014).

[16] Hu YQ Gan YQ. Development and validity verification of adolescent resilience scale. Acta Psychol Sin (Chin). (2008) 8):902–12. doi: 10.3724/SP.J.1041.2008.00902

[17] Xiao SY. The theoretical basis and research application of Social Support Rating Scale. J Clin Psychiatry (Chin). (1994) 02):98–100.

[18] Li C Huang J Cheng YC Zhang YW. Traditional chinese medicine in depression treatment: from molecules to systems. Front Pharmacol. (2020) 11:586. doi: 10.3389/fphar.2020.00586, PMID: 32457610 PMC7221138

[19] Fischer AS Ellwood-Lowe ME Colich NL Cichocki A Ho TC Gotlib IH. Reward-circuit biomarkers of risk and resilience in adolescent depression. J Affect Disord. (2019) 246:902–9. doi: 10.1016/j.jad.2018.12.104, PMID: 30795497 PMC6391738

[20] Miao RK Zhang S Li MY. Cumulative ecological risk and depression in college students: the mediating role of negative automatic thinking and the moderating role of mental resilience. Chin J Health Psychol (Chin). (2023) 31:1197–201. doi: 10.13342/j.cnki.cjhp.2023.08.016

[21] Zhou XX Ye HS. Relationship between mental resilience and parenting style and self-acceptance in adolescents with depression. J Psychiatry (Chin). (2021) 34:304–7.

[22] Peng XF Cai TT Gui TY Fu JJ. The moderating role of adolescent psychological quality in the relationship between learning stress and suicidal ideation. Chin J Ment Health (Chin). (2021) 35:919–24.

[23] McCracken LM Badinlou F Buhrman M Brocki KC. The role of psychological flexibility in the context of COVID-19: Associations with depression, anxiety, and insomnia. J Contextual Behav Sci. (2021) 19:28–35. doi: 10.1016/j.jcbs.2020.11.003

[24] Ding HS Han J Zhang ML Wang YL Lei M Wang KQ. The relationship between depressive symptoms and childhood trauma and psychological resilience in adolescents. Chin J Ment Health (Chin). (2017) 31:798–802.

[25] Yin XY Liu M Lin RZ. The influence of parental psychological distress on adolescent academic burnout: the mediating role of parental educational participation and parent-child affinity. Chin J Clin Psychol (Chin). (2022) 30:605–8. doi: 10.16128/j.cnki.1005-3611.2022.03.021

[26] Harper JM Padilla-Walker LM Jensen AC. Do siblings matter independent of both parents and friends? Sympathy as a mediator between sibling relationship quality and adolescent outcomes. J Res Adolescence. (2014) 26:101–14. doi: 10.1111/jora.12174

[27] Cheong EV Sinnott C Dahly D Kearney PM. Adverse childhood experiences (ACEs) and later-life depression: perceived social support as a potential protective factor. BMJ Open. (2017) 7:e013228. doi: 10.1136/bmjopen-2016-013228, PMID: 28864684 PMC5588961

[28] Watanabe N Nishida A Shimodera S Inoue K Oshima N Sasaki T. Help-seeking behavior among Japanese school students who self-harm: results from a self-report survey of 18,104 adolescents. Neuropsychiatr Dis Treat. (2012) 8:561–9. doi: 10.2147/NDT.S37543, PMID: 23209369 PMC3509995

[29] Cao X Zhang LH. Self-esteem and early prevention of sexual abuse in left-behind children: the mediating role of loneliness. J Guizhou Normal Univ (Chin). (2020) 38:100–3. doi: 10.16614/j.gznuj.zrb.2020.01.016

[30] Feng J Liu HY. An empirical study on the correlation between career decision-making self-efficacy and mental health status of vocational college students. Educ Theory Pract (Chin). (2010) 30:25–7.

[31] Wu L Zhang D Cheng G Hu T. Bullying and social anxiety in chinese children: moderating roles of trait resilience and psychological suzhi. Child Abuse Negl. (2018) 76:204–15. doi: 10.1016/j.chiabu.2017.10.021, PMID: 29132045

[32] Han XP Geng F Liu B. Relationship between anxiety and family environment and psychological resilience in adolescents. J Clin Exp Med (Chin). (2021) 20:2652–5.

[33] Liu CH. The relationship between high school students’ social support, self-efficacy and career maturity (chinese). (Master). (2020).

[34] Wang LQ Li Q Gu C Zhu HQ. The relationship between family intimacy, adaptability and depression among adolescents in Haikou City. Chin School Health (Chin). (2017) 38:1650–2. doi: 10.16835/j.cnki.1000-9817.2017.11.015

[35] Whitlock J Lloyd-Richardson E Fisseha F Bates T. Parental secondary stress: the often hidden consequences of nonsuicidal self-injury in youth. J Clin Psychol. (2018) 74:178–96. doi: 10.1002/jclp.22488, PMID: 28493555

[36] Ma MK Zhang YL Ruan K. Relationship between family intimacy, mental toughness and non-suicidal self-injury among Zhuang adolescents in Nanning. Modern Prev Med (Chin). (2022) 49:1792–6.

[37] Yan ZQ Zeng X Zhu S Chen L. The relationship between emotional empathy and depression in adolescents: the chain mediating role of shame tendency and psychological resilience. Chin J Clin Psychol (Chin). (2022) 30:77–80. doi: 10.16128/j.cnki.1005-3611.2022.01.016

[38] Chang YL Wang X. A research on college students’ Mental toughness and employment psychology. Higher Educ Forum (Chin). (2020) 10):86–92.

[39] Yao T Li HR Fu XX Zhao SQ Li F Wu J. Structural equation model analysis on the influence of parenting style, bullying and mental toughness on mental sub-health of adolescents. J Huazhong Univ Sci Technol (Chin). (2022) 51:192–7.

